# Smooth Muscle Silent Information Regulator 1 Contributes to Colitis in Mice

**DOI:** 10.3390/ijms26051807

**Published:** 2025-02-20

**Authors:** Xiaoqin Liu, Yu Song, Mengmeng Shen, Xinlong Liu, Wendi Zhang, Haibin Jiang, Mei Han

**Affiliations:** Department of Biochemistry and Molecular Biology, College of Basic Medicine, Key Laboratory of Vascular Biology of Hebei Province, Key Laboratory of Neural and Vascular Biology of Ministry of Education, Hebei Medical University, Shijiazhuang 050017, China

**Keywords:** SIRT1, smooth muscle cells, cZFP609, HIF-1α, inflammatory bowel disease

## Abstract

Smooth muscle cells (SMCs) are an essential component of the intestine, play an important role to maintain intestine structure, and produce peristaltic and segmentation movements. The silent information regulator 1 (SIRT1) has a dual role along with possible mechanisms in the different experimental models of inflammatory bowel disease (IBD). However, very little is known about other putative roles that overexpression of SIRT1 in SMCs may have. Here, we explored the role of SMC SIRT1 in colonic mucosa regeneration and recovery after DSS-induced colitis. We showed that smooth-muscle-specific SIRT1 transgene (*Sirt1*-Tg) mice have abnormal baseline intestinal architecture. The overexpression of SIRT1 impaired the recovery after DSS-induced injury. Furthermore, we showed that smooth-muscle SIRT1 affected the intestinal epithelial regeneration after damage by releasing cZFP609, which inhibited the hypoxia-inducible factor (HIF)-1α nuclear translocation. Together, we identify an important signaling axis cZFP609-HIF-1α linking SMCs and intestinal epithelium, which is involved in colitis development.

## 1. Introduction

Ulcerative colitis is a chronic inflammatory bowel disease (IBD), characterized by superficially mucosal inflammation occurring in the rectum and the colon [1,2]. Its exact etiology is still uncertain but genetic predisposition, immune dysregulation, and gut microbiota imbalance have been linked to IBD, which is thought to result from an inappropriate and continuing inflammatory response to commensal microbes in a genetically susceptible host [3,4,5]. Smooth muscle cells (SMCs) are an essential component of the intestine, which maintain intestine structure and produce peristaltic and segmentation movements [6,7,8]. The previous study showed that smooth-muscle-specific deletion of tumor suppressor genes leads to defective epithelial growth [9]. However, very little is known about other supposed roles that SMCs might have.

The silent information regulator 1 (SIRT1), an NAD+-dependent histone deacetylase, plays vital roles for mitochondrial metabolism, DNA repair, oxidative stress, and other principal biological procedures [10,11]. SIRT1 has a dual role along with possible mechanisms in the different experimental models of IBD [12]. Although there are extensive studies on the role of SIRT1 in the colonic mucosa, the effect of smooth-muscle SIRT1 on the colonic epithelial has not been well-clarified. We have demonstrated that the smooth-muscle-specific human SIRT1 transgenic (*Sirt1*-Tg) mice exhibit an attenuated phenotypic switching of vascular SMCs in response to hypoxia. Especially, *Sirt1*-Tg SMCs expressed highly and released cZFP609, which inhibited angiogenesis [13]. These findings directed us to ask whether and how *Sirt1*-Tg SMCs regulate intestinal epithelial homeostasis and regeneration after injury.

In the present study, we conducted a dextran sulfate sodium (DSS)-induced experimental colitis model using *Sirt1*-Tg mice and showed that *Sirt1*-Tg mice displayed much more severe inflammation. We identified that cZFP609 was expressed and released by the colonic SMCs (CSMCs) of *Sirt1*-Tg mice. Furthermore, the cZFP609 suppressed intestinal epithelial regeneration after DSS-induced injury via blockade of hypoxia-inducible factor (HIF)-1α nuclear translocation. Our research will provide a theoretical basis for the repair of colitis.

## 2. Results

### 2.1. Sirt1-Tg Mice Exhibit Abnormal Baseline Intestinal Architecture

**Figure 1:**
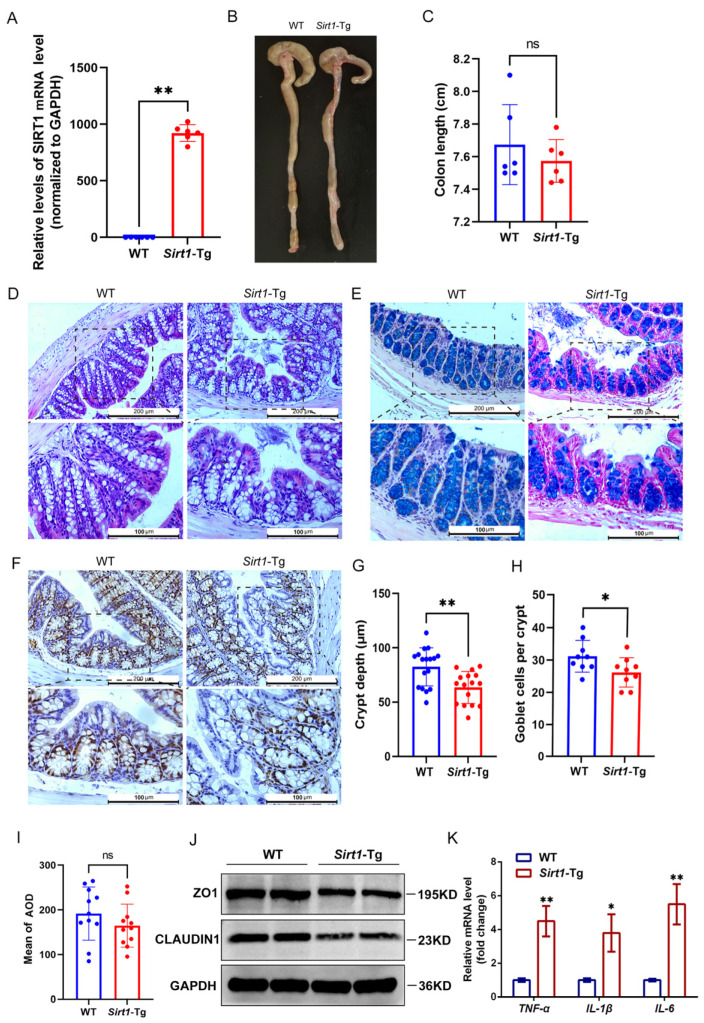
Abnormal baseline intestinal architecture in mice *Sirt1*-Tg mice compared to WT mice. (**A**) qRT-PCR for the expression of SIRT1 in mouse colon smooth muscle tissue. (**B**) Representative images showing colon length of WT and *Sirt1*-Tg mice. (**C**) Statistics of colon length of WT and *Sirt1*-Tg mice. (**D**) Representative H&E-stained images, out of three independently acquired, of colon sections from the indicated groups of mice (scale bar: 200 µm; scale bar in the enlarged image is 100 µm). (**E**) Representative Alcian-Blue-stained images, out of three independently acquired, of colon sections from the indicated groups (scale bar: 200 µm; scale bar in the enlarged image is 100 µm). (**F**) Representative photomicrographs of colonic PCNA IHC staining in each group (scale bar: 200 µm; scale bar in the enlarged image is 100 µm). (**G**) Quantified colonic crypt depth. (**H**) Quantification of the average number of goblet cells per crypt. (**I**) AOD analysis of IHC results of CLAUDIN2, *n* = 6. (**J**) Western blot detection of ZO1 and CLAUDIN1. (**K**) qRT-PCR for the expression of inflammation-related cytokines. All quantifications are represented as mean ± SD and statistical significance was assessed by two-tailed unpaired Student’s *t*-test. Actual *p*-values are indicated in each graph. * *p* < 0.05; ** *p* < 0.01; ns indicates no significant change. AOD: average optical density; GAPDH: glyceraldehyde-3-phosphate dehydrogenase.

We first verified that the mRNA of human SIRT1 was highly expressed in the colonic smooth muscle tissue of *Sirt1*-Tg mice compared to the wild-type (WT) animals (Figure 1A). There is no obvious difference in the appearance of the colon between WT and *Sirt1*-Tg mice at 8–10 weeks of age (Figure 1B,C). Notably, detailed histologic examination revealed some abnormalities in the architecture of the intestinal crypt compartments in the colon of adult *Sirt1*-Tg mice (Figure 1D,G). The number of gut epithelial goblet cells decreased in *Sirt1*-Tg mice (Figure 1E,H). However, there was no significant difference in the expression of PCNA (Figure 1F,I). Furthermore, we detected the expression of zonula occludes protein 1 (ZO1) and CLAUDIN1, and found that their expression was lower in *Sirt1*-Tg mice compared with WT (Figure 1J). Meanwhile, the expression of inflammation-related cytokines TNF-α, IL-1β, and IL-6 increased significantly in *Sirt1*-Tg mice compared with WT (Figure 1K). These data suggest that the colonic morphology was evidently altered in *Sirt1*-Tg mice and, thus, the intestinal function may also be altered.

### 2.2. Sirt1-Tg Mice Exhibit Impaired Colonic Epithelium Regeneration in DSS-Induced Colitis

**Figure 2:**
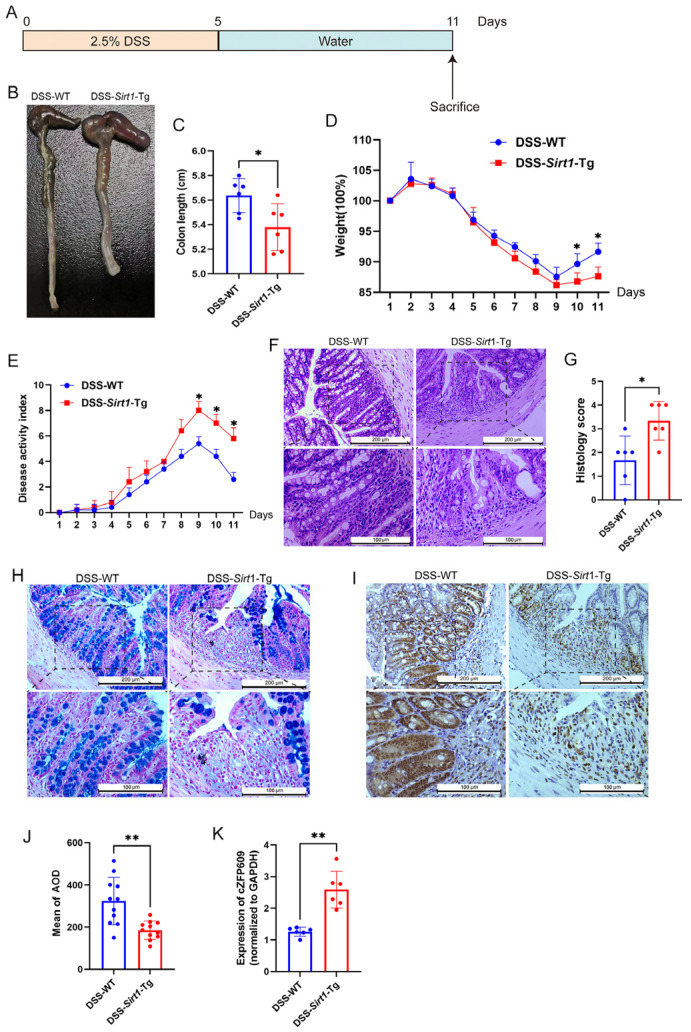
*Sirt1*-Tg mice exhibit impaired intestinal regeneration in DSS-induced colitis. (**A**) Timeline of DSS treatment. Mice were exposed to 2.5% DSS for 5 days followed by 6 days of regular water to allow for epithelial restoration. (**B**) Representative images showing colon length at end point. (**C**) Statistics of colon length of WT and *Sirt1*-Tg mice with DSS. (**D**) Weight loss relative to % to initial weight, *n* = 6 mice per genotype, determined daily and compared to the weights at the start of DSS treatment for each mouse. (**E**) Disease activity index measured daily in mice described in a during DSS treatment schedule. (**F**) Representative H&E-stained images, out of three independently acquired, of colon sections from the indicated groups of mice treated at the end of DSS administration (scale bar: 200 µm; scale bar in the enlarged image is 100 µm). (**G**) Histopathological changes in colon tissue of WT and *Sirt1*-Tg mice with DSS. (**H**) Representative Alcian-Blue-stained images, out of three independently acquired, of colon sections from the indicated groups of mice treated at the end of DSS administration (scale bar: 200 µm; scale bar in the enlarged image is 100 µm). (**I**) Representative immunohistochemistry images of the PCNA in the colon, out of three independently acquired, of colon sections from the indicated groups of mice treated at the end of DSS administration (scale bar: 200 µm; scale bar in the enlarged image is 100 µm). (**J**) AOD analysis of IHC results of PCNA, *n* = 6. (**K**) qPCR analysis of cZFP609 levels in colon tissue of mice at the end of DSS administration. Data are presented as mean ± SD of *n* = 6. All quantifications are represented as mean ± SD and statistical significance was assessed by two-tailed unpaired Student’s *t*-test. Actual *p*-values are indicated in each graph. * *p* < 0.05, ** *p* < 0.01 (two-tailed Student’s *t*-test).

To test if smooth muscle—particularly *Sirt1*-Tg smooth muscle—could play a role in intestinal injury responses, we used DSS to induce experimental colitis and compare WT to *Sirt1*-Tg mice (Figure 2A). After 5 days, DSS was replaced by regular drinking water to allow for intestinal repair, which initiates rapidly and requires reprogramming of the intestinal epithelium. On day six, we found that both WT and *Sirt1*-Tg mice displayed body weight loss and increased disease activity index, which reached their peak on the ninth day of the experiment, indicating that damage was induced equally (Figure 2B–E). At the end of 11 days, WT mice exhibited the appearance of a normal colon, reduced weight loss, and a high disease activity index, suggesting ameliorated colitis. Nevertheless, *Sirt1*-Tg mice showed significant colon shortening, continuous weight loss, and a higher disease activity index compared to WT mice (Figure 2B–E). Furthermore, other disease features were also exacerbated in *Sirt1*-Tg mice compared to WT mice. H&E analysis further revealed a larger area of ulcerated mucosa and lower presence of epithelial crypts in *Sirt1*-Tg mice compared to WT mice (Figure 2F,G). In addition, we found a significant reduction in the proliferative epithelium in *Sirt1*-Tg mice at the end point (Figure 2H–J), as determined by PCNA immunohistochemistry, suggesting that epithelial reprogramming toward a reparative state was inhibited in *Sirt1*-Tg mice.

### 2.3. Sirt1-Tg CSMC-Derived cZFP609 Inhibits the Proliferation of Caco-2 Cells via Inhibiting HIF1α Nuclear Translocation

**Figure 3:**
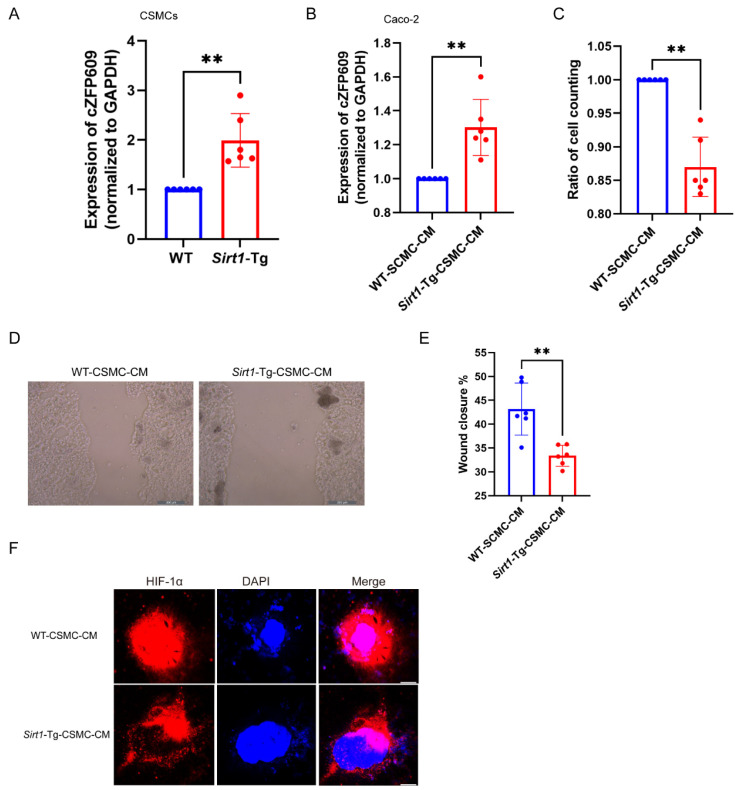
The conditioned medium (CM) of *Sirt1*-Tg CSMCs inhibits the proliferation of Caco-2 cells in vitro. (**A**) qRT-PCR of cZFP609 expression in CSMCs from WT or *Sirt1*-Tg mice treated with TNF-α for 24 h. (**B**) qRT-PCR of cZFP609 expression in Caco-2 cells incubated with the TNF-α-induced CSMC conditioned medium (CM) for 24 h and exposed to hypoxia. (**C**) Ratio of cell counting of Caco-2 cells incubated with the TNF-α-induced CSMC (CM) for 24 h and exposed to hypoxia. (**D**,**E**) Migration of Caco-2 cells was assessed using a scratch wound assay. Caco-2 cells were incubated with the TNF-α-induced CSMC exosomes (CM) for 24 h and exposed to hypoxia treated with TNF-α. (Scale bar: 200 µm.) Data are presented as mean ± SEM. (**F**) Immunofluorescent confocal microscopy of HIF1α nuclear translocation in the Caco-2 cells, Scale bars: 25 μm. Bar graphs show mean ± SEM. Student’s *t*-test or one-way ANOVA was used. ** *p* < 0.01 versus the corresponding control.

**Figure 4:**
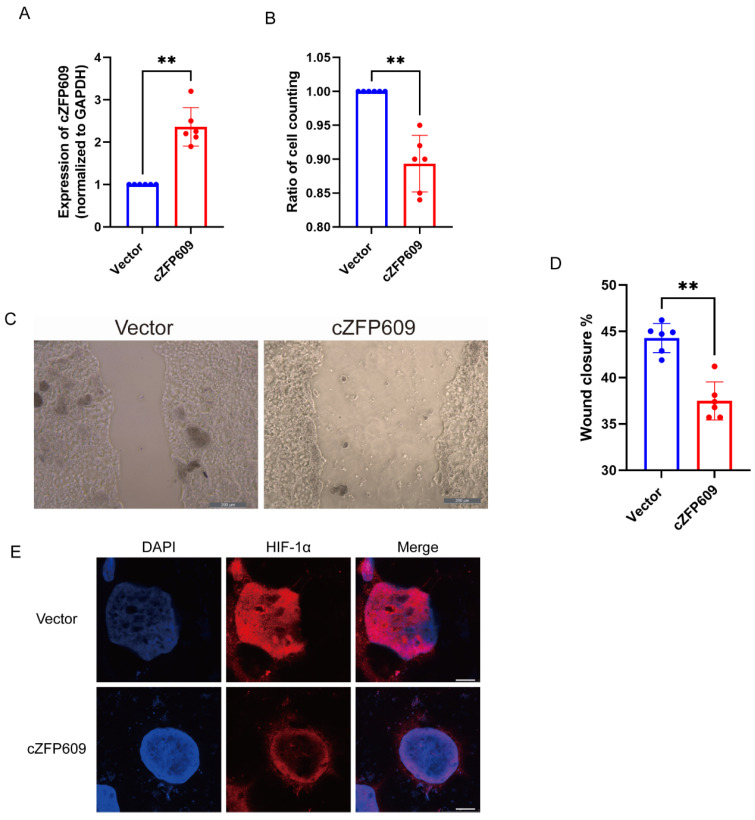
cZFP609 inhibits proliferation in Caco-2 cells via inhibiting HIF1α nuclear translocation. (**A**) qRT-PCR of cZFP609 expression in Caco-2 cells treated with vector or cZFP609. (**B**) Ratio of cell counting of Caco-2 cells treated with vector or cZFP609. (**C**,**D**) Migration of Caco-2 cells was assessed using a scratch wound assay. Caco-2 cells treated with vector of cZFP609. (Scale bar: 200 µm). Data are presented as mean ± SEM. (**E**) Immunofluorescent confocal microscopy of HIF1α nuclear translocation in the Caco-2 cells. (Scale bars: 25 μm). Bar graphs show mean ± SEM. Student’s *t*-test or one-way ANOVA was used. ** *p* < 0.01 versus the corresponding control.

We have demonstrated that the vascular SMCs of *Sirt1*-Tg mice highly expressed cZFP609, which inhibits angiogenesis after ischemia via reprogramming endothelial cells. We showed that the expression of cZFP609 was higher in DSS-induced colitis of *Sirt1*-Tg mice than in WT mice (Figure 2K). Moreover, a higher expression of cZFP609 was detected in cultured *Sirt1*-Tg CSMCs and their CM-treated Caco-2 cells (Figure 3A,B). It has been known that the gastrointestinal tract subsists in a state of physiologically low oxygen levels. To determine how *Sirt1*-Tg CSMCs inhibited colonic epithelial regeneration following DSS-induced injury observed in the model, we used the conditioned media (CM) of WT and *Sirt1*-Tg CSMCs induced with TNF-α to treat human colon adenocarcinoma Caco-2 cells. Hypoxia-induced proliferation and migration were inhibited in *Sirt1*-Tg CM-treated Caco-2 cells (Figure 3B–E). HIF-1α in epithelial cells is vital for epithelial regeneration following DSS-induced injury [14]. We found that *Sirt1*-Tg CM abolished HIF-1α nuclear translocation in Caco-2 induced by hypoxia (Figure 3F). Furthermore, we transfected cZFP609 to Caco-2 cells (Figure 4A) and found that the overexpression of cZFP609 resulted in reduced activity of the proliferation and migration in hypoxia-induced Caco-2 cells (Figure 4B–D). Importantly, cZFP609 overexpression negated HIF-1α nuclear translocation induced by hypoxia (Figure 4E). Collectively, these data suggest that cZFP609 mediated the inhibitory effect of *Sirt1*-Tg CSMCs on Caco-2 proliferation.

### 2.4. cZFP609 Aggravates DSS-Induced Injury and Impairs Epithelial Regeneration In Vivo

**Figure 5:**
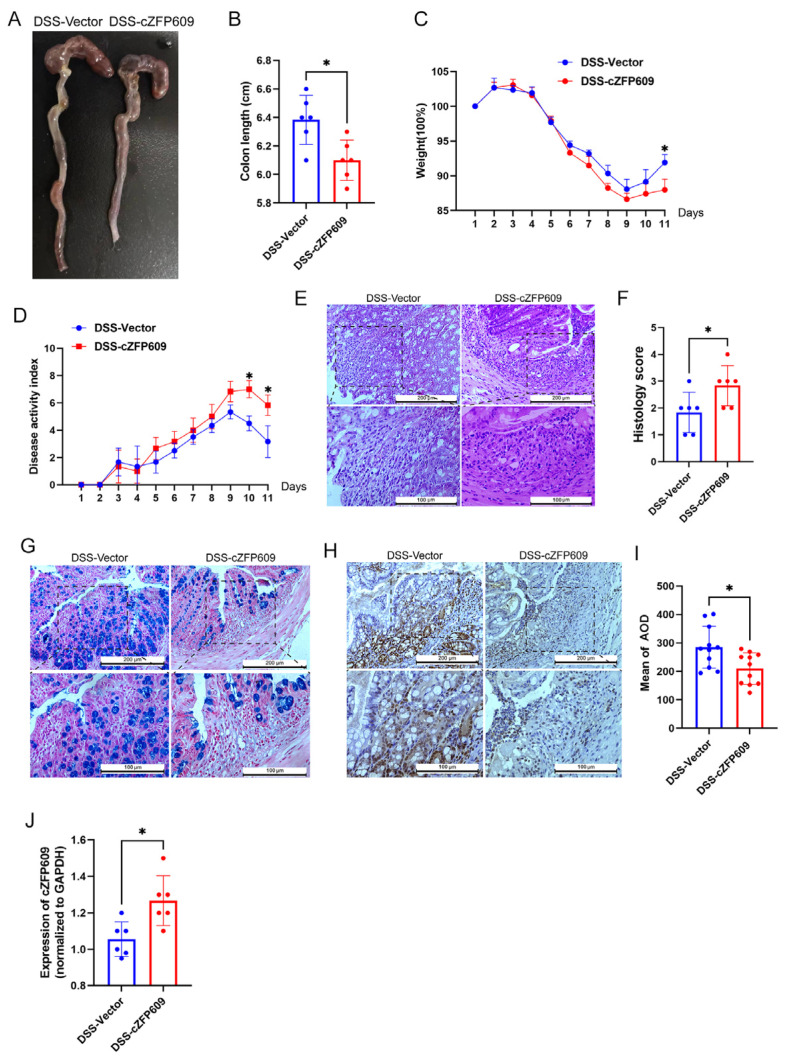
cZFP609 inhibits endothelial regeneration after DSS-induced colitis. (**A**) Macroscopic colon appearance of tail-injected WT mice treated with DSS-induced colitis. (**B**) The colon length of vector and cZFP609 tail-injection mice. (**C**) Weight loss relative to % to initial weight, *n* = 6 mice per group, determined daily and compared to the weights at the start of DSS treatment for each mouse. (**D**) Disease activity index measured daily in tail-injection mice described during the DSS treatment schedule. (**E**) Representative H&E-stained images, out of three independently acquired, of colon sections from the indicated groups of mice treated at the end of DSS administration (scale bar: 200 µm; scale bar in the enlarged image is 100 µm). (**F**) Histopathological changes in colon tissue of WT and *Sirt1*-Tg mice with DSS. (**G**) Representative Alcian-Blue-stained images, out of three independently acquired, of colon sections from the indicated groups of mice treated at the end of DSS administration (scale bar: 200 µm; scale bar in the enlarged image is 100 µm). (**H**) Representative immunohistochemistry images of the PCNA in the colon, out of three independently acquired, of colon sections from the indicated groups of mice treated at the end of DSS administration (scale bar: 200 µm; scale bar in the enlarged image is 100 µm). (**I**) AOD analysis of IHC results of PCNA, *n* = 6. (**J**) qPCR analysis of cZFP609 levels in colon tissue of tail-injection treated mice at the end of DSS administration. All quantifications are represented as mean ± SD and statistical significance was assessed by two-tailed unpaired Student’s *t*-test. Actual *p*-values are indicated in each graph. * *p* < 0.05.

To confirm whether cZFP609 leads to impaired colonic epithelial regeneration following DSS-induced injury, cZFP609 plasmid was injected into WT mice treated with 2.5% DSS for 5 days and then administrated by regular drinking water. We determined the expression of cZFP609 was up-regulated in cZFP609 plasmid injection mice at the end of the 11 days (Figure 5A). cZFP609-administrated mice exhibited significant colon shortening, weight loss, and higher disease activity index compared to control mice after removal of the DSS for 6 days (Figure 5B–E). The H&E analysis further revealed a larger area of ulcerated mucosa and decreased number of goblet cells (Figure 5F–H), accompanied by a significant reduction in PCNA expression in cZFP609-overexpressed mice compared to control mice (Figure 5I,J), as indicated by the immunohistochemistry suggesting epithelial reparative dysfunction.

## 3. Discussion

In the current study, we evaluated the effect of smooth muscle SIRT1 on intestinal mucosal integrity and immune homeostasis using genetically modified mice by DSS treatment, a common approach to induce experimental colitis. The model revealed smooth-muscle SIRT1 driving mechanisms, including inflammatory cytokine release, severe mucosa damage, and the loss of goblet cells, suggesting mucosal immune imbalance and epithelial reparative dysfunction. We showed that CSMC SIRT1 negatively regulates colonic epithelium recovery after DSS-induced injury. CSMC-derived cZFP609 could be delivered to colonic epithelial cells and attenuated the proliferation via inhibiting HIF-1α nuclear translocation in response to hypoxia. These findings allowed us to develop therapeutic interventions to mitigate SIRT1 intestinal toxicity [15].

SIRT1 was reported to protect against oxidative stress [16] and plays an important role in protection against colitis [17]. Clinical and experimental studies have proven that SIRT1 is down-regulated in UC, and SIRT1 activators have the ability to improve colitis symptoms [12,18]. Losasso et al. demonstrated that mice with an intestinal-specific SIRT1 deficiency have more Paneth and goblet cells, thus protecting the mice from colitis [19]. In the present study, we confirmed the decreased goblet cells in *Sirt1*-Tg mice. Colonic epithelium requires rapid renewal and replenishment at the base of the crypt after injury [20]. However, the effect of the SMC SIRT1 overexpression on colon epithelial proliferation and renewal is not clear. According to the previously reported results, SMC-specific SIRT1 overexpression delayed blood flow recovery and attenuated endothelial angiogenic function following hindlimb ischemia [13]. In this study, we used a DSS-induced mouse model to explore the effect of smooth-muscle-specific SIRT1 overexpression on colonic epithelial proliferation and renewal in mice with colitis. SIRT1 overexpression in SMC inhibits the recovery of colonic epithelium after DSS-induced injury. We thought that cZFP609 may function through a similar mechanism in colonic epithelial repair as both vascular endothelium and intestinal epithelium belong to barrier tissues, which share some functions and pathological processes [21]. Given that SIRT1 is well-known for its antioxidant and anti-inflammatory properties—via affecting multiple biological processes by deacetylating a variety of proteins including histones and non-histone proteins—promoting SIRT1 function is commonly used for the treatment of inflammatory diseases [22]. The clinical significance of this study lies in indicating the possible adverse effects caused by over-activation of SIRT1 and highlighting that SIRT1-targeted therapies should be conducted in a cautious approach for clinical applications.

CircRNA is a class of non-coding RNAs that forms a covalently closed continuous loop, which is conserved and stable [23]. CircRNAs have regulatory roles on mRNAs, non-coding RNAs, DNAs, and proteins, as well as various biological processes [24,25]. Based on our previous research, cZFP609 significantly increased in *Sirt1*-Tg VSMCs and was released via exosomes [13]. In the present study, we showed that the expression of cZFP609 significantly increased in *Sirt1*-Tg CSMCs. CSMC-derived cZFP609 was delivered into colonic epithelial cells that expressed low cZFP609 and acted as an HIF-1α sequestering agent to block its nuclear translocation. cZFP609 disturbed hypoxia-induced reprogramming of growth signals. The results of the present study reveal that SMC-specific SIRT1 negatively regulates the colonic epithelium regeneration after injury, resulting in a delayed recovery following DSS-induced colitis. Mechanically, the CSMC-derived cZFP609 could be delivered to the intestinal epithelial cells and attenuate the regeneration via blockade of HIF-1α nuclear translocation.

These results have clear implications for the functions of SIRT1 overexpression in intestinal smooth muscle. Studies have proved that mice with an intestinal-specific SIRT1 deficiency have more Paneth and goblet cells [19]. A shortened colon length, increased DAI, and damaged colon tissue are the characteristic symptoms of colitis. We find an important role for smooth-muscle SIRT1 in vivo. We show that *Sirt1*-Tg mice have more secretion of cZFP609 in DSS-induced mouse colitis, thus, they detained HIF-1α in the cytoplasm.

There are several limitations in the present study. First, we did not conduct direct inhibition experiments to validate that cZFP609 is a key negative regulator of colonic epithelial repair. Silencing or pharmacologically inhibiting cZFP609 in *Sirt1*-Tg mice or in vitro intestinal organoids should be conducted to provide critical evidence to confirm its causal role in intestinal mucosal damage repair. Next, in the current study, we cannot validate whether SIRT1 deletion mitigated intestinal mucosal inflammation in vivo. A future study should make efforts to clarify its effects on IBD development by specific knockdown or using a SIRT1 knockout mouse line. Finally, DSS primarily disrupts the epithelial barrier and induces acute inflammation but lacks the complexity of chronic or immune-driven colitis. A comparison to other models, such as TNBS or genetically modified mice, would help contextualize the strengths and limitations of using DSS.

## 4. Materials and Methods

### 4.1. Animals and Ethics Statement

All animal procedures conformed to the *Guide for the Care and Use of Laboratory Animals* published by the US National Institutes of Health (NIH Publication, 8th Edition, 2011) and were approved by the Institutional Animal Care and Use Committee of Hebei Medical University. SMC-specific *Sirt1*-Tg mice [26] were kindly gifted by Dr. Hou-Zao Chen of the Chinese Academy of Medical Sciences and Peking Union Medical College, China. We used a total of 36 8-week-old male mice, including 18 in the WT group and 18 in the *Sirt1*-Tg group. All mice were housed in a specific pathogen-free environment under a 12 h/12 h light–dark cycle and fed rodent diet ad libitum.

### 4.2. DSS Mouse Model of Colitis

It is well-recognized that DSS is directly toxic to colonic epithelium leading to severe illness [27]. DSS-induced colitis and a regeneration mouse model were established according to a published procedure [28]. Briefly, mice were administered with water containing 2.5% (*w*/*v*) DSS (MW 36–50 KDa, MP Biomedical LLC, Solon, OH, USA) for 5 consecutive days, and then followed by 6 days of normal drinking water, which was defined as regeneration after colitis. During the experiment, the body weight and disease activity index of the mice were monitored every day. For the in vivo cZFP609 overexpression group, male, 8-week-old C57BL/6J mice were injected with cZFP609 plasmid or control plasmid. The constructs in these experiments included 10 µg of cZFP609 plasmid or an empty vector, which were diluted in 0.9% NaCl. Plasmids were rapidly (less than 10 s) injected into the mouse’s lateral tail vein at 48 h intervals during DSS-induced colitis for the regeneration mouse model [29,30].

### 4.3. Assessment of Colitis Severity

Disease activity index (DAI) scores were used to comprehensively evaluate the severity of colonic inflammation from three aspects, including weight loss, stool consistency, and fecal bleeding, which were blindly assessed [31]. The scoring systems were defined as follows: weight loss: 0 = no loss, 1 = 1–5%, 2 = 5–10%, 3 = 10–20%, 4 = 20%+; stool: 0 = normal, 2 = loose stool, 4 = diarrhea; bleeding: 0 = no blood, 2 = Hemoccult positive (Hemoccult II; Beckman Coulter, Fullerton, CA, USA), and 4 = gross blood (blood around anus). DAI scores were measured on all 5 + 6 days of DSS treatment. Macroscopic damage scores were blindly scored using a previously published scoring system for DSS-induced colitis [32]. The animals were excluded if they prematurely died as this prevented the harvest of biological samples. The mice were sacrificed and colon tissues were collected for various analyses. In addition, the histological scores of mice were assessed by two pathologists in a double-blind manner. The severity of colitis was macroscopically scored based on colonic bleeding, fecal bleeding, loosening of stool consistency, and signs of rectal bleeding.

### 4.4. Assessment of Histological Score

All trimmed rectums were fixed in 10% neutral buffered formalin. After paraffin embedding, 5-mm-thick sections were prepared. Representative sections were stained with hematoxylin and eosin (H&E) for examination under a light microscope. Histological score was assessed by inflammation severity. The villus height and crypt length were measured using Image-Pro Software (version plus 6.0, Media Cybernetics, Inc., Bethesda, MD, USA) based on photomicrographs obtained by microscopy. Each score was defined as follows: for histological changes, 0 = normal colonic mucosa; 1 = crypt damage less than 1/3; 2 = crypt damage less than 1/3–2/3; 3 = mucosal erosion; and 4 = mucosal erosion or ulcer with significant infiltration of inflammatory cells [33]. Apart from that, the colon was stained with Alcian Blue (AB) to determine the number of goblet cells.

### 4.5. Immunohistochemistry (IHC)

IHC was performed on paraffin-embedded sections. The colon tissue sections of the mice were deparaffinized, rehydrated, and antigen-retrieved for 15 min in sodium citrate at room temperature. Subsequently, the endogenous peroxidase activity was blocked using 3% hydrogen peroxide solution and the sections were blocked with 3% bovine serum albumin (BSA). Afterward, the colon sections were incubated with primary antibody PCNA (1:200, sc-56, Santa Cruz, CA, USA) overnight at 4 °C and then labeled with HRP-conjugated antibody at room temperature for 1 h. PBS was used as the negative control instead of primary antibody to ensure the specific binding of antibodies to the target protein. Stained sections were analyzed and captured under a light microscope (Nikon Corporation, Tokyo, Japan).

### 4.6. Cell Culture and Conditioned Medium Collection

Primary CSMC culture was performed using a method previously described [34]. Briefly, the smooth muscle layer was separated from the colon of WT and *Sirt1*-Tg mice, minced with scissors, then incubated for 30–45 min at 37 °C in 1 mL of digestion solution A [0.7 mg/mL papain, 1.0 mg/mL albumin, 1 mg/mL DTT in isolation medium (60 mmol/L NaCl, 85 mmol/L L-glutamic acid, 5.6 mmol/L KCl, 2.0 mmol/L MgCl_2_, 10 mmol/L HEPES, pH 7.4)]. After centrifugation at 600× *g*, collected cells were incubated for 15–30 min at 37 °C in 1 mL of digestion solution B (1.0 mg/mL collagenase, 1.0 mg/mL hyaluronidase, 1.0 mg/mL albumin). Then, cells were suspended by gentle pipetting, collected by centrifugation at 800× *g*, and resuspended in Dulbecco’s modified eagle medium (DMEM) supplemented with 4500 mg/L D-glucose, 10% inactivated fetal bovine serum (Gemini Bioproducts, Woodland, CA, USA), and 1% penicillin–streptomycin (Invitrogen, Carlsbad, CA, USA). Cells were plated at a density of 2 × 10^4^ cells/cm^2^ and maintained at 37 °C in a humidified, 5% CO_2_ atmosphere. Passages 4 to 10 of cells were used in the experiments. CSMCs were cultured in 10-cm-diameter culture dishes until they reached 90% confluence and were then stimulated with TNF-α (20 ng/mL). Twenty-four hours later, CSMCs were washed with PBS to remove the cytokines and were then cultured in FBS-free medium for 12 h. The WT and *Sirt1*-Tg CSMC conditioned medium (CSMC-CM) was collected and centrifuged at 300× *g* for 5 min, the supernatants were then passed through a 0.22 µm (Millipore, Boston, MA, USA) filter to remove cellular debris and large vesicles. The medium was then stored at −80 °C for further use.

Caco-2 cell line was purchased from Shanghai Suer Biotechnology Co., Ltd. (Shanghai, China). Cells were cultured in Dulbecco’s modified eagle medium (DMEM) supplied with 10% heat-inactivated fetal bovine serum and 1% penicillin–streptomycin. Cells were maintained in a humidified incubator with 5% CO_2_ at 37 °C. When cells were 90% confluent, the cells were digested in 0.25% trypsin with 0.53 mM EDTA and further passaged. Then, the cells were used for the experiment in the logarithmic phase.

### 4.7. Proliferation Assay

Caco-2 cells were seeded at a density of 5 × 10^4^ cells/well in DMEM containing 10% heat-inactivated fetal bovine serum and 1% penicillin–streptomycin into a 24-well plate. The next day, the cells were treated with WT or *Sirt1*-Tg CSMC-CM and incubated at 37 °C for 24 h under hypoxia. Cell proliferation was evaluated by cell counting using a Neubauer-improved counting chamber (Marienfeld, Berlin, Germany).

### 4.8. Scratch Wound Assay

The scratch wound assay was conducted as previously described [35]. Briefly, when Caco-2 cells formed a 100% confluent monolayer, a scratch was made in each culture using a disposable pipette tip (200 µL), and the cells were washed 3 times with PBS. Then, the cells were treated with WT or *Sirt1*-Tg CSMC-CM and incubated at 37 °C for 24 h under hypoxia. The wound area was photographed using a Motic AE 2000 inverted microscope (Motic Corporation, Xiamen, China) 24 h after CM treatment. The wound area was then measured using ImageJ software (version 1.8.0, NIH, Bethesda, MD, USA).

### 4.9. Immunofluorescence Staining

Caco-2 cells grown on cover slips were fixed in 4% paraformaldehyde for 15 min, permeabilized with PBS supplemented with 0.1% Triton X-100, and then blocked using 5% normal goat serum in PBS. The cells were then incubated with mouse anti- HIF1α (1:100, GTX640664, GeneTex, Irvine, CA, USA) antibody at 4 °C overnight. The cells were washed three times with PBS and incubated with fluorescein-conjugated secondary antibodies (FITC goat anti-rabbit IgG, KPL, Gaithersburg, MD, USA) for 1 h at room temperature. Images were acquired using a fluorescence microscope (Olympus, Tokyo, Japan).

### 4.10. Quantitative Reverse-Transcription Polymerase Chain Reaction (RT-qPCR)

The tissues of the mouse colon were homogenized using a TissueLyser (Qiagen, Venlo, The Netherlands). Total RNAs were isolated using TRIzol reagent (Life Technologies, Carlsbad, CA, USA). To quantify the amount of mRNA and circRNA, cDNAs were synthesized using the M-MLV First Strand Kit (Life Technologies, Carlsbad, CA, USA), and quantitative PCRs were performed using SYBR Green qPCR SuperMix-UDG (Life Technologies, Carlsbad, CA, USA). For quantification, all RNA expression was normalized to the amount of GAPDH using the 2^−ΔΔCt^ method. PCR primer sequences are in Table 1.

### 4.11. Plasmid Construction

To construct cZFP609 expression plasmids, mouse cZFP609 cDNA was synthesized by Sangon Biotech (Shanghai, China) and cloned into pcD-ciR vector (Geneseed Biotech, Guangzhou, China). The pcD-ciR vector contained a front circular frame and a back circular frame.

### 4.12. Cell Transfection

Cells in the exponential phase of growth were plated in six-well plates at 2 × 10^5^ cells/well and cultured for 24 h (three wells for each group). The pcD-ciR-cZFP609 plasmids were transfected into cells at concentration of 50 nmol/L using Lipofectamine R 2000 (Invitrogen, Carlsbad, CA, USA) for 24 h, according to the manufacturer’s instructions. The cells were harvested at 48 h post-transfection for further analysis. All the experiments were repeated three times.

### 4.13. Statistical Analysis

Each treatment group consisted of 5–6 individual mice. All statistical analyses were carried out using the GraphPad Prism 5 statistics package. Data were first tested for normality to determine whether to use a parametric or non-parametric ANOVA. In the case of ANOVA, Bonferroni’s post hoc test was used. In the case of non-parametric ANOVA, Dunn’s post hoc test was used. The *p*-values less than 0.05 were deemed to be statistically significant. Data are presented as mean ± standard error of the mean (S.E.M).

## 5. Conclusions

In summary, we demonstrated that cZFP609 was highly expressed in the CSMCs of *Sirt1*-Tg mice, which may be associated with increased susceptibility to colitis via suppression of HIF-1α nuclear translocation. This study identifies an important signaling axis cZFP609-HIF-1α—linking SMCs and intestinal epithelium—which is involved in colitis development.

## Figures and Tables

**Table 1:** PCR primer sequence.

Primer Sequence		
Mouse ZFP609	Forward	GGCCACTAAAGAAAGTCAAGTCTG
	Reverse	GGACATCTTAGAGTCAACGTCCC
Human SIRT1	Forward	TGTTTCATGTGGAATACCTGA
	Reverse	TGAAGAATGGTCTTGGATCTT
Mouse GAPDH	Forward	TGGATTTGGACGCATTGGTC
	Reverse	TTTGCACTGGTACGTGTTGAT

## Data Availability

The data used and/or analyzed during this article are available from the corresponding author upon reasonable request.

## Abbreviations

The following abbreviations are used in this manuscript:

SMCs	Smooth muscle cells
SIRT1	Silent information regulator 1
IBD	Inflammatory bowel disease
HIF	Hypoxia-inducible factor
DSS	Dextran sulfate sodium
CSMCs	Colonic smooth muscle cells
CM	Conditioned media
